# Mixture of Anthraquinone Sulfo-Derivatives as an Inexpensive Organic Flow Battery Negolyte: Optimization of Battery Cell

**DOI:** 10.3390/membranes12100912

**Published:** 2022-09-21

**Authors:** Mikhail Petrov, Dmitry Chikin, Lilia Abunaeva, Artem Glazkov, Roman Pichugov, Alexey Vinyukov, Irina Levina, Mikhail Motyakin, Yaroslav Mezhuev, Dmitry Konev, Anatoly Antipov

**Affiliations:** 1 EMCPS Department, Mendeleev University of Chemical Technology of Russia, 125047 Moscow, Russia; 2 Institute for Problems of Chemical Physics, Russian Academy of Sciences, 142432 Chernogolovka, Russia; 3 Emanuel Institute of Biochemical Physics, Russian Academy of Sciences, 119334 Moscow, Russia; 4 Semenov Federal Research Center for Chemical Physics, Russian Academy of Sciences, 119991 Moscow, Russia; 5 Department of Biomaterials, Mendeleev University of Chemical Technology of Russia, 125047 Moscow, Russia

**Keywords:** redox flow batteries, organic redox flow batteries, negolyte, anthraquinone derivative, AQDS, cyclic voltammetry, sulfonation, electrochemical performance, battery cell, symmetric cell cycling, Luggin capillary

## Abstract

Anthraquinone-2,7-disulfonic acid (2,7-AQDS) is a promising organic compound, which is considered as a negolyte for redox flow batteries as well as for other applications. In this work we carried out a well-known reaction of anthraquinone sulfonation to synthesize 2,7-AQDS in mixture with other sulfo-derivatives, namely 2,6-AQDS and 2-AQS. Redox behavior of this mixture was evaluated with cyclic voltammetry and was almost identical to 2,7-AQDS. Mixture was then assessed as a potential negolyte of anthraquinone-bromine redox flow battery. After adjusting membrane-electrode assembly composition (membrane material and flow field)), the cell demonstrated peak power density of 335 mW cm^−2^ (at SOC 90%) and capacity utilization, capacity retention and energy efficiency of 87.9, 99.6 and 64.2%, respectively. These values are almost identical or even higher than similar values for flow battery with 2,7-AQDS as a negolyte, while the price of mixture is significantly lower. Therefore, this work unveils the promising possibility of using a mixture of crude sulfonated anthraquinone derivatives mixture as an inexpensive negolyte of RFB.

## 1. Introduction

Redox flow batteries (RFB) are a promising type of secondary chemical power sources where the electricity is stored in form of chemical energy of two electrolytes [1]. One of them is a negolyte circulating in negative half-cell and entering into redox reactions at anode and another is a posolyte circulating in the positive half-cell and reacting at the cathode. RFB is considered as a promising high-capacity energy storage technology operating in the electrical grid in order to smooth out the imbalance between a variable power supply and demand [1,2,3]. Such devices are becoming especially relevant in the context of rejecting harmful carbon energy sources in favor of alternative energy characterized by an irregular electricity generation.

There are many types of RFBs using different electrolytes [4]. In terms of technology maturity and effective implementation in energy networks, all-vanadium RFB (VRFB) occupies a special place. VRFBs utilize electrolytes based on vanadium salts in aqueous solutions of sulfuric, hydrochloric and other acids [5,6,7,8]. They have several advantages over other RFB types, but at this technological level their wide distribution is hindered by a high cost of energy storage [9]. Despite the high prices of membranes, their contribution to the capital cost of VRFB is lower than electrolytes by up to 37% versus 50%, respectively [10]. This is due to a very volatile and high price of vanadium determined by different resource constraints [11]. For this and other reasons, various alternative electrolytes are being considered for use in redox flow batteries.

One of the options is various organic compounds, which can be obtained from abundant raw precursors. They provide great opportunities for controlling properties via chemical structure modification and can theoretically offer high energy densities [4]. Due to rapid charge transfer kinetics, high solubility and suitable redox potentials anthraquinone derivatives attract particular attention. Among them 2,7-AQDS is distinguished due to high chemical stability during redox reactions and other advantages [12,13,14]. In 2014, anthraquinone-bromine RFB (ABRFB) was presented, which used an aqueous sulfuric acid solution of 2,7-AQDS as a negolyte and molecular bromine aqueous solution as a posolyte [15]. This battery demonstrated specific power of 0.6 W cm^−2^ and relatively high discharge capacity retention of 99.2% at a current density of 200 mA cm^−2^, which opened up good opportunities for its practical application in the future.

In subsequent works, the ABRFB concept was intensively developed. Specific power was increased to 1 W cm^−2^ [16] and conditions for cyclic charge-discharge tests were optimized in such a way that energy efficiency (EE) of 88% and discharge capacity retention of 99% [17] were achieved. The prospects of using this concept with differential pH were demonstrated in [18]. In addition, AQDS/H_2_AQDS is actively combined with other redox pairs besides Br^−^/Br_2_ [19,20,21,22], as well as other various anthraquinone derivatives which are considered as negolytes in RFB [23,24,25,26,27,28,29,30,31,32,33,34]. Moreover, 2,7-AQDS application is not limited to RFBs, but also used in other devices; for example, fuel cells [35], microbial fuel cells [36] and hybrid energy storage devices [37,38,39].

An estimated capital cost of ABRFB is equal to 117 $/kWh, which is significantly lower than for VRFB (173 $/kWh) and just slightly more than for iron-chromium RFB (101 $/kWh) [40], known as one of the cheapest RFBs [41]. However, as far as we know, none of the commercial ABRFBs have been installed. This is primarily explained by the high cost of pure 2,7-AQDS contributing up to 60% of ABRFB total cost [40]. Also, the price of 2,7-AQDS rises dramatically if total production falls below 1000 tons per year [42].

One promising opportunity to reduce this cost is to use a mixture of 2,7-AQDS with other electroactive anthraquinone derivatives (anthraquinone-2,6-disulfonic acid 2,6-AQDS and monosulfonated derivative 2-AQS) [43,44], instead of pure 2,7-AQDS, which is originally separated from this mixture by additional procedures [45]. This synthesis uses commodity chemicals such as anthraquinone and oleum and resulting price of organic electroactive materials obtained this way is estimated at $1 to $4 per kg [42] which is no more than $40 per kilowatt-hour, i.e., сomparable or less than for VRFBs or even Li-ion batteries [45].

However, to date the possibility of using crude anthraquinone sulfo-derivatives mixture (ASM) as a negolyte in RFB has not been systematically assessed [46]. The redox behavior of such a system was only briefly characterized using the cyclic voltammetry (CV) method [15]. Apart from widespread 2,7-AQDS, other ASM components (2,6-AQDS and 2-AQS) were tested separately as negolytes of RFB [22,23]. The only exception is recent research by Mazur et al. focused mainly on chemical stability of ASM components during redox reactions [47] and confirming the fundamental possibility of using ASM as RFB negolyte. However, key parameters determining capabilities to use the ASM in RFB, namely discharge power, current density, energy efficiency, were not evaluated.

Our work is intended to eliminate this gap. We synthesized ASM using aforementioned synthetic approach and described ASM composition with use of NMR spectroscopy and other auxiliary methods. Further, we systematically studied ASM redox behavior using CV and performed a series of experiments, where ASM was used as a negolyte of ABRFB. During these measurements the discharge cell design and operating conditions were adjusted to achieve maximum discharge power. Also, we provide systematic analysis of the ABRFB performance (power density, energy efficiency, capacity utilization and capacity retention) using AS mixture and 2,7-AQDS as a negolyte, discuss the prospects of practical use of the AS mixture as a negolyte of ABRFB and outline ways to optimize the composition of the AS mixture by changing synthesis conditions.

## 2. Materials and Methods

### 2.1. Electrolyte Preparation

To prepare 2,7-AQDS in acidic form, anthraquinone-2,7-disulfonic acid disodium salt (>97.0%, TCI, Tokyo, Japan) in aqueous solution was passed through a pretreated ion exchange column with KU-2-8 cation exchanger (“AZOT”, Sievierodonetsk, Ukraine). After reaching neutral pH at the outlet, the resulting solution was evaporated on a rotary evaporator to desired concentration. Sulfuric acid (95–95%, Sigma Tek, Khimki, Russia) was used as a supporting electrolyte. In case of 2,6-AQDS (disodium salt, >98.0%, Sigma-Aldrich, St. Louis, Missouri, USA) and 2-AQS (monosodium salt, >98%, «Sigma-Aldrich», USA) the same procedure was used.

Synthesis of ASM containing various anthraquinone sulfonated derivatives was performed according to the method of anthraquinone sulfonation [43,44,48]. Oleum (20–24%, TK ANT, Saint-Petesburg, Russia) was poured into a round-bottomed flask placed in HJ-6A mantle heater (Alkhitech, Moscow, Russia), heating and stirring were switched on, and then a weighed portion of anthraquinone (98%, Acros Organics, Geel, Belgium) was added when the temperature of reaction mixture reached 70 °С. The ratio by weight of oleum: anthraquinone was in the range between 1:3 and 1:4. Then, the mixture was heated to 160–170 °C and maintained at this temperature for 2 h. After that, the synthesis products were slowly cooled with a large amount of distilled water (triple distilled water, UD-3015, ULAB, Saint-Petersburg, Russia). As a result, a solution of mono- and disulfo-substituted anthraquinones in weakly concentrated sulfuric acid with density of 1.129 g mL^−1^ was obtained. After a while, a slight precipitation was observed in the resulting reaction mixture. For all experiments, a supernatant was used as an electrolyte.

### 2.2. Electrochemical Measurements

Cyclic voltammetry (CV) measurements were performed in a three-electrode cell under inert atmosphere (argon > 99.998%, BK Group, Moscow, Russia). The background electrolyte was 1 M sulfuric acid. The working electrode was 3 mm glassy carbon electrode. Before experiments, the electrode surface was polished on abrasive paper with a different grain size to avoid the electrode’s sphericity. Ag/AgСl (saturated KCl solution, 0.197 V vs SHE) was used as a reference electrode; platinum foil was used as a counter electrode. The measurements were carried out on Autolab 302N potentiostat (Metrohm, Herisau, Switzerland).

### 2.3. Battery Assembly

MEA with a 4 cm^2^ electrode surface area was made according to [49]. Nafion 211 and 117 (Chemours, Wilmington, Delaware, USA) and GP-IEM-103 (Liaoning Grepalofu New Energy Co., Taiping Town, China) were used as proton exchange membranes. Three sheets of carbon paper electrodes (SGL39AA, SGL Carbon, Wiesbaden, Germany) were used as electrodes in both half-cells. The combination of pressed graphite foil sheets (thickness 600 µm, Unihimtek, Podolsk, Russia) formed a 3D structure of serpentine and flow-through flow field types. Viton™ fluoroelastomer was used as material of sealing gaskets. Both electrolytes were kept in an argon atmosphere (>99.998%, BK Group, Russia).

The internal resistance of cells as measured by electrochemical impedance spectroscopy (EIS) and corresponded to a real part of impedance at zero imaginary component at high frequencies. Measurements were performed using Autolab 302N potentiostat, which set a 10 mV sinusoidal perturbation superimposed onto OCV, and frequency ranged from 1 Hz to 50 kHZ.

Modification of the described cell was used during experiments with Lugging capillary. In a modified cell flow field gaskets were made from Teflon^TM^ sheets and carbon felt Sigracell GFD 4.6 (SGL Carbon, Wiesbaden, German) was used as electrodes. Membrane capillaries made of Nafion 117 stripes placed between electrode gaskets and membrane acted as reference electrodes which provide an ability to separate anodic and cathodic polarizations [50,51].

### 2.4. Flow Cell Experiments

Unless otherwise mentioned, the following conditions were used: 25 mL of negolyte (2,7-AQDS or AS mixture), 50 mL of posolyte (0.5 M Br_2_/3.5 M HBr, the discharge capacity of posolyte is intentionally excessive in order to prevent the appearance of corrosive molecular bromine even at fully charged state of the battery [52]), serpentine flow field, Nafion 211 membrane, electrolyte flow rate 100 mL min^−1^.

The measurements were carried out on a potentiostat-galvanostat P150-X (Elins, Chernogolovka, Russia). The polarization curves were measured by method of chronopotentiometry with charge compensation in a potentiostatic mode for SOC 100% and galvanostatic mode for SOC 90%. The polarization curves at SOC 75% and 50% were measured by applying alternating values of charge/discharge current density with fixed time intervals. The open-circuit voltage (OCV) of the cell was measured simultaneously with charging to SOC 100%. For this measurement, we used an additional potentiometric cell of our in-house design which was introduced into electrolyte lines and operated as an inline voltmeter. Cyclic tests were conducted at current density 100 mA cm^−2^ and 200 mA cm^−2^ in a voltage range from 0 to −1.3 V for 10 charge/discharge cycles. The obtained dependences were used to calculate coulombic, voltaic and energy efficiency (CE, VE and EE) as well as capacity utilization and retention in accordance with generally accepted formulas [53].

### 2.5. NMR Spectroscopy and Other Characterization Techniques

ASM were dried on a rotary evaporator. The resulting brown-orange products were dissolved in D_2_O and ^1^H NMR and ^13^C NMR spectra were recorded on a Bruker Avance 500 spectrometer (Bruker, Zurich, Switzerland).

Redox titration was carried out by gradually adding NaOH using phenolphthalein as indicator. Coulometric measurements were carried out in AQDS-H_2_ cell with the design similar to ABRFB discharge cell. A glassy carbon plate with shading was used as a positive electrode, a Freudenberg H23C8 carbon paper with a deposited Pt/C catalyst (with platinum loading 1 mg cm^−2^) as a negative electrode, and Nafion 211 as a proton exchange membrane. AQDS electrolysis reduction was carried out with a potential difference of 0.5 V until current reached stationary value attributed to the crossover rate.

Optical and near-UV spectra were recorded on a UV/VIS/NIR Spectrometer Lambda 750 (Perkin Elmer, Waltham, MA USA). The optical path length was 10 mm. The measurements were carried out in the wavelength range from 190 to 900 nm. The test solutions were diluted with distilled water; reference spectra—distilled water.

Long-term stability of ASM was evaluated by symmetric cell cycling [54]. ASM diluted to 0.02 M by 2.2 M H_2_SO_4_ was used as both negolyte and posolyte, that were pumped through the discharge cell described above. An initial SOC of both electrolytes was adjusted to 50% by a potentiostatic electrolysis via H_2_/H^+^ redox-pair. Charge–discharge test was carried out in a potentiostatic regime by repetitive allying of voltage of +0.2 V and 0.2 V. Polarization was changed after the current was reduced to 2 mA. The volume exceeded that of the other by 4 times (20 mL vs 80 mL).

## 3. Results and Discussions

### 3.1. Composition of ASM

**Scheme 1:**
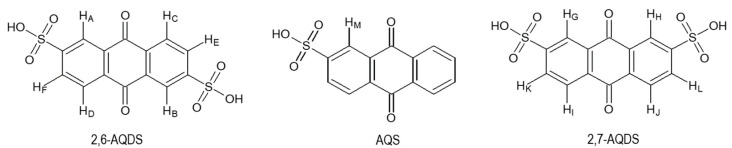
The chemical structure of 2,7-AQDS, 2,6-AQDS and AQS.

The sulfonation of anthraquinone with oleum allows us to obtain the mixture of various sulfonated derivatives of anthraquinone (2,7-AQDS, 2,6-AQDS and AQS; see Scheme 1) and unreacted anthraquinone dissolved in weakly concentrated sulfuric acid [43,44,47,55]. The exact composition can be accomplished by controlling the conditions of synthesis: temperature, concentration of the starting reagents, time, and the presence of various additives. The composition of ASM used in our experiments was determined via three different methods: nuclear magnetic resonance spectroscopy, redox titration and coulometry.

**Figure 1:**
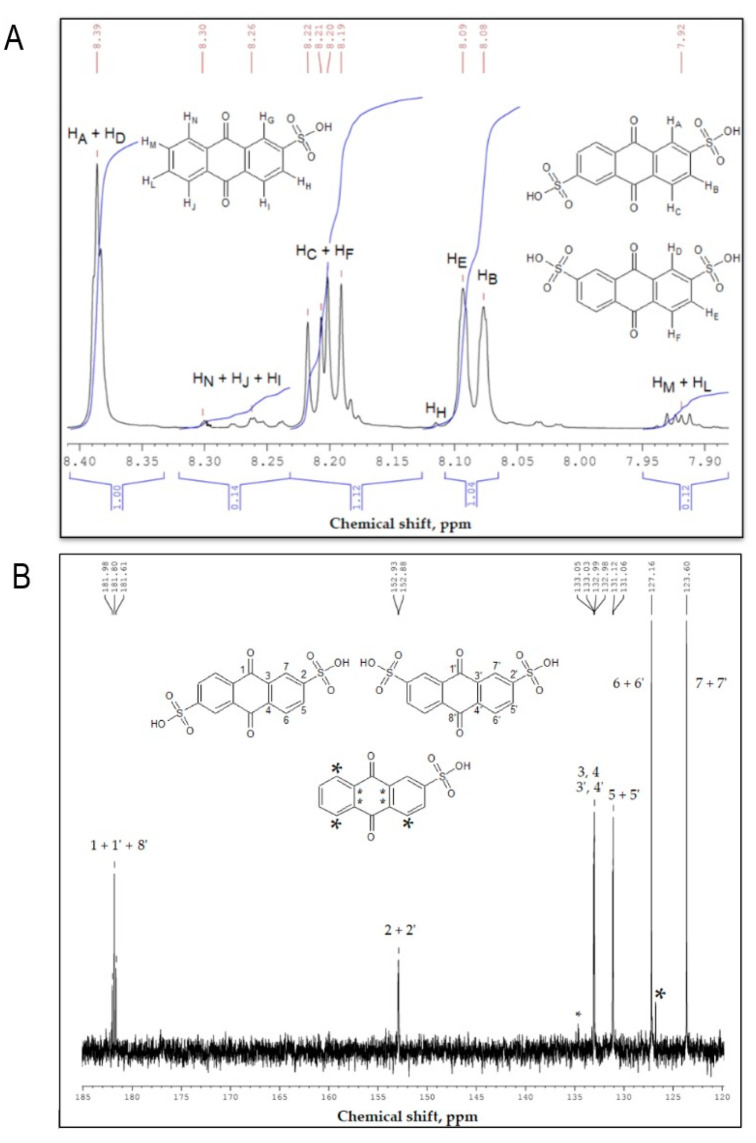
NMR (500 MHz, D_2_O) spectra of AS mixture. (**А**) ^1^Н NMR (**B**) ^13^C NMR.

The ^1^H NMR spectrum of anthraquinone sulfonation products (Figure 1A) shows a number of signals indicating the formation of a mixture containing 2,7-AQDS, 2,6-AQDS and 2-AQS. The almost identical ratio of the integrated intensities of protons (H_E_ + H_B_)/(H_C_ + H_F_)/(H_A_+H_D_) is consistent with the molecular formulas of 2,7-AQDS and 2,6-AQDS. As far as the ratio of H_E_/H_B_ is equal to 0.533/0.489, the molar ratio of 2,6-AQDS to 2,7-AQDS is 1.13.

**Figure 2:**
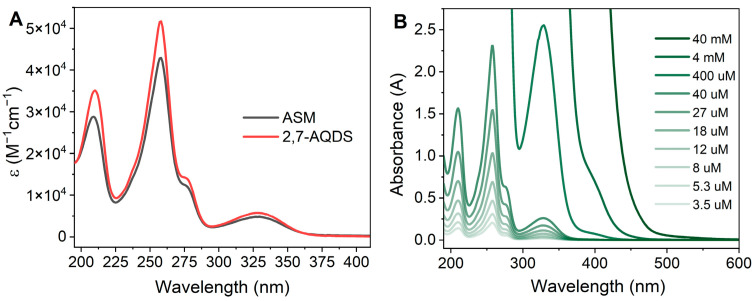
(**A**) Molar extinction coefficients of 2,7-AQDS and ASM (**B**) Optical and near-UV absorption spectra of 2,7-AQDS diluted by deionized water, optical path length—1 cm.

The content of AQS in ASM is significantly lower. A multiplet with a chemical shift of 7.92 ppm could be ascribed to H_M_ and H_L_ protons of AQS. Apparently, the signals of protons H_G_ and partially H_I_ and H_H_ are overlapped by 2,6-AQDS and 2,7-AQDS signals. Thus, a reliable estimation of AQS content could only be made using the integrated intensities of H_M_ and H_L_, which is equal to 0.12 and 0.06 per AQS proton, respectively. Therefore, the molar fractions of 2,7-AQDS, 2,6-AQDS and 2-AQS are 50.2%, 44.4% and 5.4%, respectively. The ^1^H NMR spectroscopy data are consistent with the assignment of bands in the ^13^С NMR spectrum and literature data [23,56]. Since the discrepancies between the chemical shifts of 2,7-AQDS and 2,6-AQDS are negligible, a series of intensive paired singlets can be observed (Figure 2B).

The AQS content among the sulfonation products is insufficient to observe signals from all carbon nuclei. However, the signals of two groups of AQS carbon atoms with similar chemical shifts are distinguishable (Figure 1B). Thus, it can be concluded that after the formation of AQS, repeated sulfonation occurs leading to almost equal amount of 2,6-AQDS and 2,7-AQDS isomers with a slight predominance of the latter. This is consistent with a slightly lower electron density at position 6 compared to position 7 for AQS due to the manifestation of a mesomeric effect of the already existing sulfo group.

Additional measurements were carried out to numerically estimate the content of various components in the mixture. Acid-base titration gave an estimate for the total concentration of the monosulfonated AQS and the doubled concentration of the disulfonic acid derivatives 2,6- and 2,7-AQDS as well as the sulfuric acid itself. At the same time, coulometric titration allows to estimate the total concentration of all sulfonated anthraquinone derivatives without the contribution of the sulfuric acid. On the basis of the experiments performed, the following composition of ASM was obtained: 0.19 M 2,7-AQDS, 0.16 M 2,6-AQDS, and 0.02 M AQS against the background 2.18 M H2SO4.

It should be noted that for interpreting coulometry data we assumed that during the AQDS/AQS reduction two electrons are consumed for each molecule. There is evidence that, due to the formation of intermolecular complexes, each AQDS/AQS molecule can obtain a maximum of 1.5-e during the reduction process [56,57]. However, in our experiments the typical two-electron transfer was observed to be in agreement with other reported data [17,58].

Also, the obtained ASM composition is confirmed by optical and near-UV absorption spectra (see Figure 2). The characteristic AQDS absorption peaks lie at 210, 260, 275 and 330 nm [59,60]. Using the absorption spectra for 2,7-AQDS solutions at various dilutions (Figure 2B), the molar extinction coefficient at wavelength 260 nm was calculated to be ε = 51,150 M cm^−1^. This value is in good agreement with other reported data where it ranges from 39,000 M cm^−1^ [59] to 55,700 M cm^−1^ [60].

It is known that the optical absorption spectra of 2,7-AQDS and AQS do not differ dramatically from each other [59]. Similarly, the absorption spectra of 2,6-and 2,7-AQDS isomers should also be similar since all discrepancies between these derivatives comes down to the position of sulfo groups. Therefore, molar absorbance spectra of ASM and 2,7-AQDS should be quite similar, both quantitatively and qualitatively. We estimated the molar extinction coefficients of ASM based on overall concentration given above (0.37 M) and obtained a sufficiently good agreement between ASM and 2,7-AQDS spectra (Figure 2A).

### 3.2. Electrochemical Behavior

**Figure 3:**
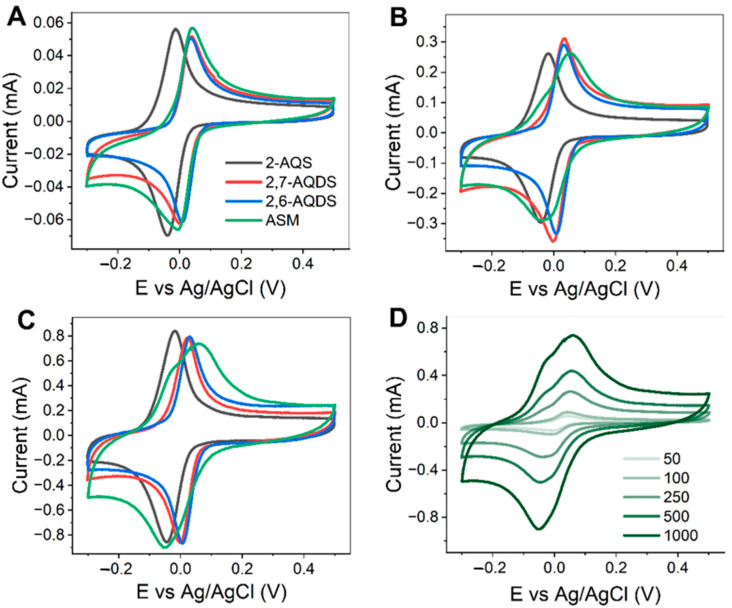
Cyclic voltammograms for 2,7-AQDS, 2,6-AQDS, 2-AQS and ASM. Total concentration of sulfonated derivatives—1 mM, supporting electrolyte—1 M H_2_SO_4_. Scan rate: (**А**) 50 mVs^−1^ (**B**) 250 mVs^−1^ (**C**) 1000 mVs^−1^. (**D**) represents CV of ASM for scan rate from 50 mVs^−1^ to 1000 mVs^−1^.

In order to evaluate the prospects of using ASM as an RFB negolyte, redox behavior was investigated using the CV method and compared with redox behavior of individual isomers. Figure 3A shows CV at 50 mVs^−1^. CVs of 2,7-AQDS and 2,6-AQDS are almost identical and represent pair of well-resolved redox peaks with half-wave potential E_1/2_~0.02 V vs. Ag/AgCl reference electrode (or E_1/2_ = 0.217 V vs. SHE). The peak-to-peak separation for 2,7-AQDS and 2,6-AQDS is around 30 mV, which is in good agreement with the two-electron reduction behavior of the AQDS molecule. The peak position of monosulfonated derivative shifts to the cathodic region by 45–50 mV (see Table S1 in Supplementary Materials), corresponding to the shift of AQS standard potential caused by the removal of one electron-withdrawing sulfonate groups [23]. Besides this distinction, CV of 2,7-AQDS/2,6-AQDS look similar and the general picture remains almost intact as the scan rate increases from 50 mVs^−1^ to 500 mVs^−1^ (Figure 3B,C).

The redox behavior of ASM undergoes much more noticeable changes with increasing scan rate. At 50 mVs^−1^ CV of ASM only differs from 2,7-AQDS/2,6-AQDS in a slight increase in peak-to-peak separation up to 54 mV, which indicates some minor kinetic complications. With an increase of the scan rate (Figure 3D), the picture changes significantly. The reduction peak acquires a pronounced asymmetry and shifts to the cathodic region, oxidation peak is divided into two peaks; one of them shifts to the anodic region, the other, less intense, shifts to the cathodic region. As a result, CV of ASM at increased scan rate represents a superposition of mono- and disulfonated derivatives. Also, one should note that in the case of ASM, an increase of scan rate leads to a significant increase in peak-to-peak separation and a decrease in cathodic/anodic peak current ratio, which emphasized the aforementioned kinetic complications (see Table S1).

According to CV data, the redox behavior of ASM is determined by the behavior of isomers contained. It is also characterized by some kinetic limitations, which presumably might be ascribed to the presence of different intermolecular complexes [56,57]. Regardless, ASM could still be used as a negolyte of RFB, and the question of whether it affects the RFB performance will be discussed further.

### 3.3. Cell Optimization

It is known that the design of battery cell can significantly affect the characteristics of RFB [61]. One of the key elements is semipermeable membrane which, on the one hand, should prevent mixing of negolyte and posolyte and, on the other hand, should ensure the transport of balancing counterions from one half-cell to another (in the case of ABRFB—H^+^ protons). Selection of a membrane may greatly affect the overall performance of RFB [62]. To explore this possibility the flow cell with cation-exchange membranes Nafion 211 and GP-IEM-103 were used.

Nafion 211 is one of the well-known commercial perfluorosulfonic acid membrane, which is widely used in different RFB, fuel cells, etc. As one of the thinnest membranes it was selected to estimate the limits of ABRFBs power density. GP-IEM-103 is an inexpensive perfluorosulfonic proton-exchange membrane with conductivity 0.1 S/cm and density 2 g/cm^3^, which can be also used in RFB [63]. The key difference between them is thickness, for Nafion 211 it is 25 µm, and for GP-IEM-103—75 µm. Also, these membranes are slightly differed by the swelling ratio in used organic solution (ASM in 2.2 M H_2_SO_4_), for Nafion 211 and GP-IEM-103 this value is 1.07 and 1.05, respectively. Moreover, these membranes are comparable in terms of permeability of bromine [64]—which plays a key role in the crossover during ABRFB operation.

**Figure 4:**
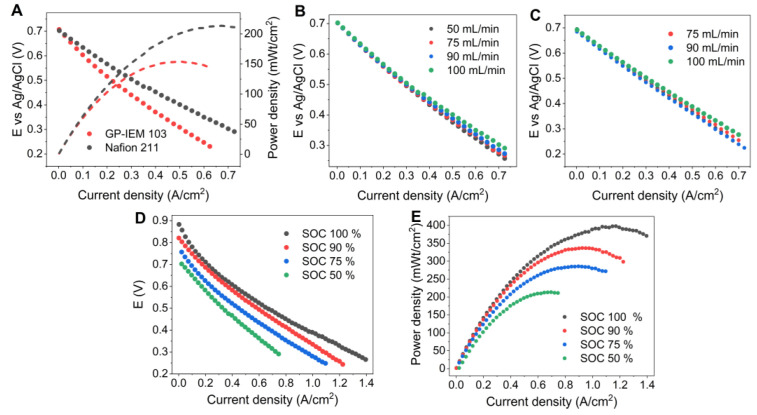
(**A**) Polarization curves (dots) and corresponding power densities (dash) of ABRFB with GP-IEM-103 and Nafion 211 membrane at SOC 50%, Flow field—serpentine, flow rate—50 mL min^−1^ (**B**,**C**) Polarization curves of ABRFB with Nafion 211 membrane, flow field—serpentine (**B**), flow-through (**C**) polarization curves at SOC 50% at different flow rates (**D**) Polarization curves of ABRFB with Nafion 211 membrane, flow field—flow through, flow rate 100 mL min^−1^, SOC changes from 50% to 100% (**E**) Corresponding power densities. ASM as a negolyte in all cases.

The internal resistance of cells with different membranes (estimated with EIS data) was different: 0.40 and 0.25 Ω cm^2^ or GP-IEM-103 and Nafion 211, respectively. Figure 4A shows polarization curves and power characteristics for ABRFBs with Nafion 211 and GP-IEM-103 membranes. From polarization curves it is seen that behavior of the cell is mainly determined by ohmic losses [65] except for the region of low current densities (up to 0.1 A cm^−2^) where activation losses dominate. From the slope of polarization curve in the linear section it is possible to estimate the discharge resistance which represents the sum of various contributions including internal and charge-transfer resistances [65]. This value was 0.52 and 0.66 Ω cm^2^ for cells with Nafion 211 and GP-IEM-103, stating that the main difference between cell discharge resistances is dictated by the internal resistance, which, in turn, governed mainly by the membrane resistance. In addition, the use of a thinner membrane may lead to a decrease of overall RFB cost [62]. Accordingly, the cell with Nafion 211 exhibited a higher discharge power and was used for further experiments.

Another useful way to influence the characteristics of ABRFB is associated with varying type of flow field and flow rate. It is known that optimal configuration of a flow field ensuring uniform electrolyte distribution over electrode surface and good wettability of electrode surface can significantly improve a performance of cell [66,67,68,69]. In this case, the geometry of a flow field should be optimized considering flow rate since a performance of cell strongly depends on this parameter and this dependence varies with a flow field type. In this work, we compared serpentine and flow-through flow field types at flow rates from 50 to 100 mL min^−1^. Figure 4B,C show corresponding polarization curves that differ from each other only by a slight decrease of discharge resistance with increase of the flow rate. Table 1 shows the power densities values calculated from polarization curve data. For the serpentine flow field, the specific power increases with the flow rate from 185 to 200 mW cm^−2^, for the flow-through type—from 194 to 214 mW cm^−2^. It is seen that a higher discharge power is demonstrated for the flow-through type at all flow rates. Therefore, for further experiments we used the flow-through geometry at flow rate 100 mL min^−1^.

Figure 4D,E show the polarization curves and power characteristics for ABRFB with an optimal cell design at various battery SOCs (100, 90, 75 and 50%). It can be seen that all curves demonstrate similar behavior. The region responsible for activation losses is observed for small current densities and becomes especially noticeable at high SOCs (90 and 100%). The remaining part of the polarization curve is controlled by the ohmic losses and the discharge resistance does not differ significantly for different SOCs. The fundamental difference between polarization curves is that they correspond to different open circuit voltages (OCV) which depend on SOC in full accordance with the Nernst equation. Accordingly, the highest discharge power is observed at SOC 100%—398 mW cm^−2^ achieved at 1200 mA cm^−2^.

For comparison, the highest discharge power of ABRFB using pure 2,7-AQDS as negolyte is 1 W cm^−2^ [16]. In that research 1 M solution of 2,7-AQDS was used while the total concentration of various sulfonated derivatives in our AS mixture is 0.36 M. Therefore, the specific power of our ABRFB can still be significantly increased with the increase of ASM concentration. In addition, in [16], the method of linear potential sweep was used to measure polarization curve and electrolytes were kept at an elevated temperature of 40 °C, which leads to overestimated power values.

**Figure 5:**
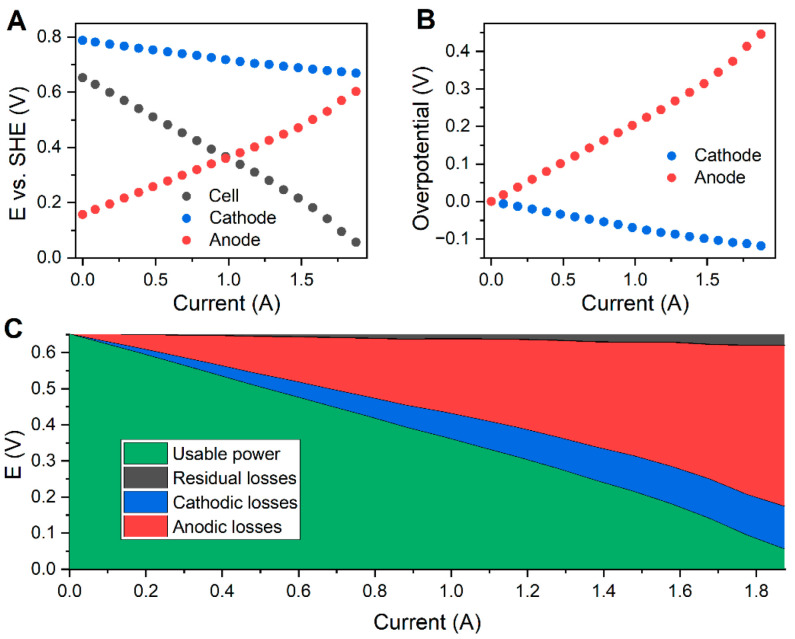
(**A**) Cell voltage, positive and negative electrode potentials (**B**) Electrode overpotentials (**C**) Polarization curves with separated losses. ABRFB using ASM as negolyte, flow field—flow through, flow rate—100 mL min^−1^, SOC 100%. Electrode—carbon felt Sigracell GFD 4.6.

To estimate whether it is possible to improve the power density of ABRFB with ASM negolyte, we measured the polarization curves of ABRFB MEA with simultaneous recording of the electrode potential using a special-designed cell with incorporated Luggin capillary. Results of the measurement are shown on Figure 5a. Corresponding overpotentials were calculated using electrode potential subtracted from zero-current potentials, i.e., electrode potential under measured OCV conditions (see Figure 5B). It can be seen that the discharge resistance of the negative electrode (ASM side) significantly exceeds that of the positive electrode (Br side) and equal 0.80 and 0.28 Ω cm^2^, respectively. Using overpotential dependencies one could separate power losses of negative electrode, positive electrode and residual constituents (Figure 5), which are mainly attributed to internal resistance (see [51] for details).

Figure 5C clearly demonstrates that negative electrode losses play a key role among all power losses. Therefore, there is still some feasibility to improve power density of ABRFM by adjusting the resistance of negative electrode. In turn, it could be achieved by an enrichment of electrode surface area and increase of electrolyte conductivity. But this issue lays beyond the scope of this research. Therefore, further we will give a more detailed analysis of our ABRFB performance which uses crude products of anthraquinone sulfonation as a negolyte in comparison with the same cell design using pure 2,7-AQDS.

### 3.4. Comparison of ASM and 2,7-AQDS

Since a pH change leads to the Nernst shift of standard redox potential and to a change in reaction mechanism [70] we used 0.4 M solution of 2,7-AQDS in 2.2 M H_2_SO_4_ as a reference negolyte—the composition close to ASM both in total concentration of anthraquinone sulfonates and sulfuric acid. Figure 5A shows the polarization curves and power characteristics of ABRFB with various negolytes at SOC 50%.

**Figure 6:**
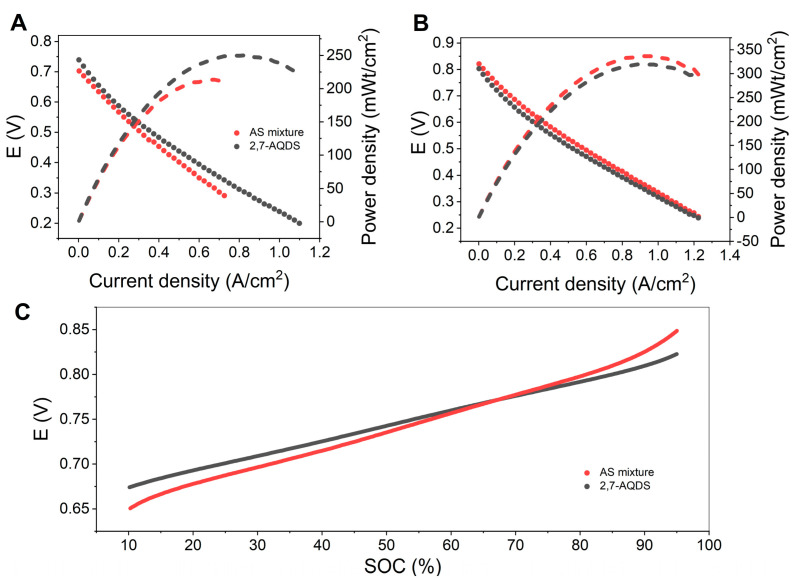
Polarization curves (dots) and corresponding power densities (dash) for ABRFB using as a negolyte ASM or 2,7-AQDS (0.4 M in 2.2 M H_2_SO_4_). Membrane—Nafion 211, Flow field—flow through, SOC—50% (**A**) and 90% (**B**) Flow rate—100 mL min^−1^ (**C**) Corresponding dependence of OCV vs. SOC.

The specific discharge power of cell using 2,7-AQDS as a negolyte is higher. At SOC 50% it is 250 mW cm^−2^ against 194 mW cm^−2^ for ASM (Figure 6A). In general, dissimilar RFB performance can be explained by various reasons: differences in charge-transfer processes (the kinetics of ongoing reactions), cell voltage, mass transport properties (mainly differences in solubility and diffusion coefficients of key components of ongoing half-reactions), reactivity of various redox forms of electroactive components, contribution of side reactions (i.e., hydrogen evolution).The most obvious reason for the discrepancy is complicated kinetics of redox reactions occurring in the ASM mixture, noted before. It is confirmed by the shape of polarization curves. The discharge resistance calculated from the slope of linear part of polarization curve for cells with ASM and 2,7-AQDS is 0.50 and 0.36 Ω cm^−2^, respectively. A similar effect was observed using more concentrated solutions of crude anthraquinone sulfonation products as a negolyte [47].

In addition, the discrepancy in discharge power can be partially explained by differences in the cell voltage (Figure 6A). It was shown that the cell voltage of ABRFB using monosulfonic AQS as a negolyte is higher than relative 2,7-AQDS cell leading to the increased discharge power [23]. In our case, the ASM contains about 5 mole percent of AQS, which could also lead to an increase in cell voltage and discharge power, but this was not observed for SOC 50%. To analyze this effect and more comprehensively compare the capabilities of using ASM and 2,7-AQDS as a negolyte, we also measured the dependences of the OCV vs SOC (see Figure 6C).

It can be seen that, at the qualitative level the dependences for ASM and 2,7-AQDS are practically identical to each other. Within the SOC range from 15 to 85%, a linear increase in OCV is observed with a sharp increase and, conversely, fall at high and small SOCs, respectively. This corresponds to similar curves for ABRFB [15,23] and suggests that the cell voltage is determined by the Nernst potentials of half-cells and electroactive compounds (sulfonated anthraquinone derivatives) do not undergo decomposition reaction during cell operation. However, on a quantitative level, the curves differ significantly since OCV of the cell using ASM increases much faster with SOC than for the reference cell with 2,7-AQDS. At SOC 20% the potential of the cell with 2,7-AQDS was higher by 16 mV, at SOC 70% they became equal and at SOC 90% OCV of the cell with AS mixture was already by 16 mV higher than for the reference cell.

This behavior may be associated with an existence of various dimers in solutions of anthraquinone derivatives and, in particular, quinhydrone complexes formed between reduced and oxidized forms of the molecule due to hydrogen bonding, as well as charge transfer and dispersion interactions [57,60]. It is shown that such complexation affects a half-cell potential and cell voltage of ABRFB [60]. On the other hand, it is known that the nature of complexation can vary greatly depending on the exact composition of medium. For example, formation of quinhydrone complexes is suppressed due to steric restrictions in the structure of anthraquinone derivative with a bulky side substituent [27], and even for the simplest 1,4-benzoquinone structure of the quinhydrone complex strongly depends on a concentration of hydroquinone, as well as size and concentration of cation [71]. Therefore, changing single 2,7-AQDS to ASM, consisting of 2,6- and 2,7-AQDS and AQS, the thermodynamic constant of the quinhydrone complex formation (or other dimers and aggregates) can change significantly, which should eventually affect the OCV(SOC) dependence.

Based on a performance comparison of ABRFB cells using 2,7-AQDS and ASM as a negolyte, we can summarize that with an increase in SOC the difference in specific power of cells will gradually disappear. Accordingly, at SOC 90% the discharge power of ABRFB cell using AS negolyte becomes even higher than that for pure 2,7-AQDS: 335 vs 320 mW cm^−2^ (see Figure 6A).

**Figure 7:**
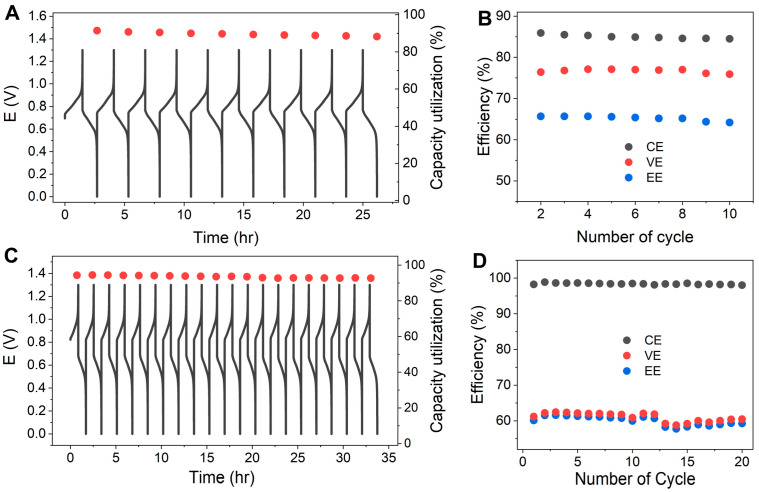
(**A**,**C**) Charge–discharge curves for constant current cycling of ABRFB at 0.1 А cm^−2^ using ASM as a negolyte; capacity utilization is indicated for each complete cycle. Flow field—flow through, Membrane—Nafion 211 (**A**) or Nafion 117 (**C**), flow rate—100 mL min^−1^, (**B**,**D**) Corresponding efficiencies for ABRFB using Nafion 211 (**B**) and Nafion 117 (**D**) membrane.

As a next step of evaluating possibilities of using ASM as an ABRFB negolyte, we carried out cyclic charge–discharge tests. Figure 7A shows the charge-discharge curves for 10 cycles (@ 100 mA cm^−2^) and corresponding capacity utilization and efficiencies. ABRFB with ASM negolyte demonstrates capacity utilization around 85%, which slightly decreases during the charge-discharge experiment. Regardless, at the end of the tenth cycle, the discharge capacity was 0.47 Ah, which, in terms of specific capacity, results in 6.3 Ah L^−1^.

Figure 7B and Table 2 show the corresponding efficiency values, as well as capacity utilization and capacity retention in comparison with ABRFB using 2,7-AQDS as a negolyte. Coulombic efficiency of the tested cells is relatively low (94–95%), compared with 98% for single 2,7-AQDS cell [17,72]. The difference can be explained by several reasons. First of all, small thickness of the Nafion 211 membrane attributes to more intense crossover of bromine-containing molecules from posolyte to negolyte [73], which, in fact, do not react with AQDS [15] but undergo reduction on the electrode surface leading to inevitable decrease of coulombic efficiency. Also, this effect is further enhanced by ionic imbalance between electrolytes due to longer duration of each cycle at lower current density.

Switching from 2,7-AQDS to ASM, the coulombic efficiency of ABRFB decreases to 84.4% on the tenth cycle. Mazur et al. analyzed the chemical stability of a similar mixture of anthraquinone sulfonated derivatives during cycling tests in a symmetric cell where the influence of bromine is excluded [48] and showed that 2,6- and 2,7-AQDS remain stable during redox reactions while AQS is gradually decomposed. This may partially explain the decrease in CE. However, due to the quite low molar content of AQS in the initial ASM, this effect will not be so noticeable. The decrease in CE is likely to be explained by complicated kinetics of redox reactions occurring in the ASM mixture which can lead to an increased role of competitive hydrogen evolution reaction. In addition, the change in CE can also be associated with characteristic differences in the OCV(SOC) dependence for 2,7-AQDS and ASM since one of the driving forces of the crossover is the difference between the nonequilibrium cell potential and OCV [17].

Nevertheless, the key numerical characteristics of cycling tests of ABRFB with ASM negolyte were comparable with results for 2,7-AQDS. After 10 charge-discharge cycles at 100 mA cm^−2^, the cell demonstrated capacity retention, capacity utilization and energy efficiency values of 99.8, 90.7 and 65.7%, respectively.

At the same time, these values can be improved by optimizing cycling conditions (current density, voltage limits), composition of the posolyte (decreasing osmotic imbalance) and using a thicker membrane. For example, changing the membrane to Nafion 117 (thickness 183 µm) and current density to 200 mA cm^−2^, the CE of cell with ASM reaches 98–99% (see Figure 7B–D and Table 2). At the same time, values of capacity utilization and capacity retention remained at the same level as for the cell with a thin membrane Nafion 211—after 20 cycles, they were 92.7 and 99.9%, respectively. However, it is obvious that transition to the thicker Nafion 117 membrane will lead to an increase in overall cell resistance and, as a consequence, to a decrease in discharge power of the cell. Therefore, a more accurate membrane choice will contribute to reaching the balance between various characteristics of the ABRFB.

In order to evaluate the redox stability of ASM an additional symmetrical cycling test was performed. Generally, during this test the same electrolyte at SOC 50 was used both as a negolyte and posolyte, which allows us to evaluate redox stability of some compound in the absence of a crossover [54]. ASM cycled for 45 cycles showed capacity fade rate of 0.015%/cycle (see Figure S1). Assuming this value to be constant, after 1000 cycles this will give a decreased total capacity of 14%.

Thus, we have shown that the crude ASM obtained as a result of a simple one-step synthesis from cheap and commodity chemicals can be used as a negolyte in ABRFB, and the main characteristics of such cells are comparable to cells using pure 2,7-AQDS. In addition, the ASM composition can be further optimized by changing the synthesis conditions (temperature, duration, concentration of the starting reagents). However, this issue requires additional systematic study considering the above-mentioned differences in solubility, standard redox potential, kinetics of ongoing reactions and redox stability of various anthraquinone sulfonated derivatives in the ASM, as well as their tendency to form complexes. In addition, the overall concentration of anthraquinone sulfonated derivatives and background acid can be changed. Together with the data presented above, it can be concluded that the use of ASM as a cheap organic electrolyte has great potential in the development of redox flow battery technology.

## 4. Conclusions

To conclude, it was shown that the treatment of anthraquinone with oleum allows us to obtain the mixture of sulfonated anthraquinone derivatives with the total concentration 0.37 M, which consists of 0.19 M 2,7-AQDS, 0.16 M 2,6-AQDS and 0.02 AQS in 2.2 M background sulfuric acid. The redox behavior of this mixture is practically identical to pure 2,7-AQDS: pair of symmetric and reversible redox peaks with E_0_ = 0.217 vs SHE is observed on the CV at 50 mV s^−1^. With the increase of scan rate reduction, peak broadens and oxidation peak is also divided into two peaks indicating kinetic limitations.

The resulting crude mixture was studied as a negolyte of ABRFB with 0.5 M Br_2_/3.5 M HBr acting as the posolyte. The design of the discharge cell (membrane, type of flow field) and flow rates were optimized to achieve the maximum specific power 398 mW cm^−2^ at SOC 100%.

The key characteristics of ABRFB utilizing crude ASM and pure 2,7-AQDS of the same concentrations were compared. Power density of the cells was comparable with even higher values for the ASM at high SOC. For instance, it was 335 vs 320 mW cm^−2^ at SOC 90% for the ASM and 2,7-AQDS, respectively. It is associated with the relatively high increment of OCV(SOC) dependence for ASM, which is presumably explained by the peculiarities of complexation in this medium. Finally, the ASM demonstrated stable operation during cycling charge–discharge tests at 100 mA cm^−2^. After 10 cycles of energy efficiency, capacity utilization and capacity retention were 65.7, 87.9 and 99.6%, respectively, which is on the same level as a cell with pure 2,7-AQDS: 67.2, 90.1 and 99.7%.

Thus, it was shown that the crude ASM could be used as the negolyte of ABRFB without the significant loss in key characteristics compared to the much more expensive pure 2,7-AQDS solution. The composition of ASM mixture can be further optimized by changing the synthesis conditions, which opens great opportunities for using these mixtures as organic electrolytes in redox flow batteries, as well as in other applications.

## Figures and Tables

**Table 1:** Peak power density of ABRFB at SOC 50% for different flow fields and flow rate. Negolyte—ASM, Membrane—Nafion 211.

Flow Rate [mL min^−1^]	Peak Power Density [mW cm^−2^]	
	Serpentine	Flow-Through
50	185	194
75	190	201
90	182	202
100	200	214

**Table 2:** Key characteristics of constant-current cycling at 0.1/0.2 А cm^−2^ using ASM/ 2,7-AQDS as a negolyte. Membrane Nafion 211/Nafion 117. All values are given for 10th cycle.

	ASM		2,7 AQDS
	Nafion 211 (@ 0.1 A cm^−2^)	Nafion 117 (@ 0.2 A cm^−2^)	Nafion 211 (@ 0.1 A cm^−2^)
Capacity Utilization, %	90.7	93.5	91.2
Capacity retention, %	99.8	99.8	99.9
Coulombic Efficiency, %	85.9	98.5	94.2
Voltaic Efficiency, %	76.4	60.9	75.8
Energy Efficiency, %	65.7	60.0	71.4

## Data Availability

Not applicable.

## Supplementary Materials

The following supporting information can be downloaded at: https://www.mdpi.com/article/10.3390/membranes12100912/s1, Figure S1: (A) Charge-discharge curves for constant current symmetrical cycling of ASM. Applied voltage–0.2. (B) Dependence of the discharge capacity on the cycle number; Table S1: Main characteristics of redox behavior obtained by CV.

## Abbreviations

RFB	redox flow battery
VRFB	vanadium redox flow battery
2,7-AQDS	9,10-anthraquinone-2,7-disulfonic acid
ABRFB	Anthraquinone-bromine
EE	energy efficiency
AQS	anthraquinone-2-sulfonic acid AQS
2,6-AQDS	9,10-anthraquinone-2,6-disulfonic acid
AS mixture	anthraquinone sulfonation mixture
CV	cycling voltammetry
SWV	square wave voltammetry
SHE	standard hydrogen electrode
SOC	state of charge
CE	coulombic (faradaic) efficiency
VE	voltaic efficiency
OCV	open circuit potential

## References

[1] Sánchez-Díez E., Ventosa E., Guarnieri M., Trovò A., Flox C., Marcilla R., Soavi F., Mazur P., Aranzabe E., Ferret R. (2021). Redox Flow Batteries: Status and Perspective towards Sustainable Stationary Energy Storage. J. Power Sources.

[2] Petrov M.M., Modestov A.D., Konev D.V., Antipov A.E., Loktionov P.A., Pichugov R.D., Kartashova N.V., Glazkov A.T., Abunaeva L.Z., Andreev V.N. (2021). Redox Flow Batteries: Role in Modern Electric Power Industry and Comparative Characteristics of the Main Types. Russ. Chem. Rev..

[3] Arenas L.F., Ponce de León C., Walsh F.C. (2019). Redox Flow Batteries for Energy Storage: Their Promise, Achievements and Challenges. Curr. Opin. Electrochem..

[4] Winsberg J., Hagemann T., Janoschka T., Hager M.D., Schubert U.S. (2017). Redox-Flow Batteries: From Metals to Organic Redox-Active Materials. Angew. Chemie. Int. Ed..

[5] Choi C., Kim S., Kim R., Choi Y., Kim S., Jung H.Y., Yang J.H., Kim H.T. (2017). A Review of Vanadium Electrolytes for Vanadium Redox Flow Batteries. Renew. Sustain. Energy Rev..

[6] Zhang Z.H., Wei L., Wu M.C., Bai B.F., Zhao T.S. (2021). Chloride ions as an electrolyte additive for high performance vanadium redox flow batteries. Appl. Energy.

[7] Yang Y., Zhang Y., Tang L., Liu T., Huang J., Peng S., Yang X. (2019). Investigations on physicochemical properties and electrochemical performance of sulfate-chloride mixed acid electrolyte for vanadium redox flow battery. J. Power Sources.

[8] Di Noto V., Vezzu K., Crivellaro G., Pagot G., Sun C., Meda L., Rutkowska I.A., Kulesza P.J., Zawodzinski T.A. (2022). A general electrochemical formalism for vanadium redox flow batteries. Electrochim. Acta.

[9] Schmidt O., Melchior S., Hawkes A., Staffell I. (2019). Projecting the Future Levelized Cost of Electricity Storage Technologies. Joule.

[10] Minke C., Kunz U., Turek T. (2017). Techno-Economic Assessment of Novel Vanadium Redox Flow Batteries with Large-Area Cells. J. Power Sources.

[11] Ciotola A., Fuss M., Colombo S., Poganietz W.R. (2021). The Potential Supply Risk of Vanadium for the Renewable Energy Transition in Germany. J. Energy Storage.

[12] Wedege K., Dražević E., Konya D., Bentien A. (2016). Organic Redox Species in Aqueous Flow Batteries: Redox Potentials, Chemical Stability and Solubility. Sci. Rep..

[13] Kwabi D.G., Ji Y., Aziz M.J. (2020). Electrolyte Lifetime in Aqueous Organic Redox Flow Batteries: A Critical Review. Chem. Rev..

[14] Er S., Suh C., Marshak M.P., Aspuru-Guzik A. (2015). Computational Design of Molecules for an All-Quinone Redox Flow Battery. Chem. Sci..

[15] Huskinson B., Marshak M.P., Suh C., Er S., Gerhardt M.R., Galvin C.J., Chen X., Aspuru-Guzik A., Gordon R.G., Aziz M.J. (2014). A Metal-Free Organic-Inorganic Aqueous Flow Battery. Nature.

[16] Chen Q., Gerhardt M.R., Hartle L., Aziz M.J. (2016). A Quinone-Bromide Flow Battery with 1 W/Cm 2 Power Density. J. Electrochem. Soc..

[17] Chen Q., Eisenach L., Aziz M.J. (2016). Cycling Analysis of a Quinone-Bromide Redox Flow Battery. J. Electrochem. Soc..

[18] Khataee A., Wedege K., Dražević E., Bentien A. (2017). Differential PH as a Method for Increasing Cell Potential in Organic Aqueous Flow Batteries. J. Mater. Chem. A.

[19] Lee W., Permatasari A., Kwon B.W., Kwon Y. (2019). Performance Evaluation of Aqueous Organic Redox Flow Battery Using Anthraquinone-2,7-Disulfonic Acid Disodium Salt and Potassium Iodide Redox Couple. Chem. Eng. J..

[20] Lee W., Permatasari A., Kwon Y. (2020). Neutral PH Aqueous Redox Flow Batteries Using an Anthraquinone-Ferrocyanide Redox Couple. J. Mater. Chem. C.

[21] Cao J., Zhu Z., Xu J., Tao M., Chen Z. (2017). Nitrogen-Doped Porous Graphene as a Highly Efficient Cathodic Electrocatalyst for Aqueous Organic Redox Flow Battery Application. J. Mater. Chem. A.

[22] Yang B., Hoober-Burkhardt L., Krishnamoorthy S., Murali A., Prakash G.K.S., Narayanan S.R. (2016). High-Performance Aqueous Organic Flow Battery with Quinone-Based Redox Couples at Both Electrodes. J. Electrochem. Soc..

[23] Gerhardt M.R., Tong L., Gómez-Bombarelli R., Chen Q., Marshak M.P., Galvin C.J., Aspuru-Guzik A., Gordon R.G., Aziz M.J. (2017). Anthraquinone Derivatives in Aqueous Flow Batteries. Adv. Energy Mater..

[24] Geysens P., Evers J., Dehaen W., Fransaer J., Binnemans K. (2020). Enhancing the Solubility of 1,4-Diaminoanthraquinones in Electrolytes for Organic Redox Flow Batteries through Molecular Modification. RSC Adv..

[25] Xia L., Huo W., Gao H., Zhang H., Chu F., Liu H., Tan Z. (2021). Intramolecular Hydrogen Bonds Induced High Solubility for Efficient and Stable Anthraquinone Based Neutral Aqueous Organic Redox Flow Batteries. J. Power Sources.

[26] Zhang S., Li X., Chu D. (2016). An Organic Electroactive Material for Flow Batteries. Electrochim. Acta.

[27] Zhu Y., Li Y., Qian Y., Zhang L., Ye J., Zhang X., Zhao Y. (2021). Anthraquinone-Based Anode Material for Aqueous Redox Flow Batteries Operating in Nondemanding Atmosphere. J. Power Sources.

[28] Kwabi D.G., Lin K., Ji Y., Kerr E.F., Goulet M.A., De Porcellinis D., Tabor D.P., Pollack D.A., Aspuru-Guzik A., Gordon R.G. (2018). Alkaline Quinone Flow Battery with Long Lifetime at PH 12. Joule.

[29] Hu B., Luo J., Hu M., Yuan B., Liu T.L. (2019). A PH-Neutral, Metal-Free Aqueous Organic Redox Flow Battery Employing an Ammonium Anthraquinone Anolyte. Angew. Chem..

[30] Caram J.A., Banera M.J., Suárez J.F.M., Mirífico M.V. (2017). Electrochemical Behaviour of Anthraquinone Dyes in Non Aqueous Solvent Solution: Part I. Medium Effect on the Electrochemical Behaviour. Electrochim. Acta.

[31] Wu M., Jing Y., Wong A.A., Fell E.M., Jin S., Tang Z., Gordon R.G., Aziz M.J. (2020). Extremely Stable Anthraquinone Negolytes Synthesized from Common Precursors. Chem.

[32] Guiheneuf S., Lê A., Godet-Bar T., Chancelier L., Fontmorin J.M., Floner D., Geneste F. (2021). Behaviour of 3,4-Dihydroxy-9,10-Anthraquinone-2-Sulfonic Acid in Alkaline Medium: Towards a Long-Cycling Aqueous Organic Redox Flow Battery. ChemElectroChem.

[33] Ruan W., Mao J., Chen Q. (2021). Redox Flow Batteries toward More Soluble Anthraquinone Derivatives. Curr. Opin. Electrochem..

[34] Lima A.R.F., Pereira R.C., Azevedo J., Mendes A., Sérgio Seixas de Melo J. (2021). On the Path to Aqueous Organic Redox Flow Batteries: Alizarin Red S Alkaline Negolyte. Performance Evaluation and Photochemical Studies. J. Mol. Liq..

[35] Preger Y., Johnson M.R., Biswas S., Anson C.W., Root T.W., Stahl S.S. (2020). Anthraquinone-Mediated Fuel Cell Anode with an Off-Electrode Heterogeneous Catalyst Accessing High Power Density When Paired with a Mediated Cathode. ACS Energy Lett..

[36] Martinez C.M., Zhu X., Logan B.E. (2017). AQDS Immobilized Solid-Phase Redox Mediators and Their Role during Bioelectricity Generation and RR2 Decolorization in Air-Cathode Single-Chamber Microbial Fuel Cells. Bioelectrochemistry.

[37] Santos M.S.S., Peixoto L., Azevedo J., Monteiro R.A.R., Dias-Ferreira C., Alves M.M., Mendes A. (2020). Microbially-Charged Electrochemical Fuel for Energy Storage in a Redox Flow Cell. J. Power Sources.

[38] Wei Z., Almakrami H., Lin G., Agar E., Liu F. (2018). An Organic-Inorganic Hybrid Photoelectrochemical Storage Cell for Improved Solar Energy Storage. Electrochim. Acta.

[39] Li W., Fu H.C., Li L., Cabán-Acevedo M., He J.H., Jin S. (2016). Integrated Photoelectrochemical Solar Energy Conversion and Organic Redox Flow Battery Devices. Angew. Chem. Int. Ed..

[40] Darling R.M. (2022). Techno-economic analyses of several redox flow batteries using levelized cost of energy storage *Curr*. Opin. Chem. Eng..

[41] Sun C., Znahg H. (2021). Review of the Development of First-Generation Redox Flow Batteries: Iron-Chromium System. ChemSusChem.

[42] Dieterich V., Milshtein J.D., Barton J.L., Carney T.J., Darling R.M., Brushett F.R. (2018). Estimating the Cost of Organic Battery Active Materials: A Case Study on Anthraquinone Disulfonic Acid. Transl. Mater. Res..

[43] Crossley M.L. (1915). The separation of mono-β-, 2,6- and 2,7-sulfonic acids of anthraquinone. J. Am. Chem. Soc..

[44] Anello L.G., Berenbaum M.B., Peterson J.O., Sukornick B., Sogn A.W. (1978). Sulfonation of anthraquinone in sulfur dioxide solvent. US Patent.

[45] Gregory T.D., Perry M.L., Albertus P. (2021). Cost and Price Projections of Synthetic Active Materials for Redox Flow Batteries. J. Power Sources.

[46] Fontmorin J.-M., Guiheneuf S., Godet-Bar T., Floner D., Geneste F. (2022). How anthraquinones can enable aqueous organic redox flow batteries to meet the needs of industrialization. Curr. Opin. Colloid Interface Sci..

[47] Mazúr P., Charvát J., Mrlík J., Pocedič J., Akrman J., Kubáč L., Řeháková B., Kosek J. (2021). Evaluation of Electrochemical Stability of Sulfonated Anthraquinone-Based Acidic Electrolyte for Redox Flow Battery Application. Molecules.

[48] Rogers D.G. (1934). Process of Sulphonating Anthraquinone and Its Derivatives. U.S. Patent.

[49] Pichugov R.D., Konev D.V., Petrov M.M., Antipov A.E., Loktionov P.A., Abunaeva L.Z., Usenko A.A., Vorotyntsev M.A. (2020). Electrolyte Flow Field Variation: A Cell for Testing and Optimization of Membrane Electrode Assembly for Vanadium Redox Flow Batteries. Chempluschem.

[50] Modestov A.D., Konev D.V., Antipov A.E., Vorotyntsev M.A. (2019). Hydrogen-Bromate Flow Battery: Can One Reach Both High Bromate Utilization and Specific Power?. J. Solid State Electrochem..

[51] Loktionov P., Bocharova A., Konev D., Modestov A., Pichugov R., Petrov M., Antipov A. (2021). Two-Membrane Acid-Base Flow Battery with Hydrogen Electrodes for Neutralization-to-Electrical Energy Conversion. ChemSusChem.

[52] Petrov M.M., Konev D.V., Kuznetsov V.V., Antipov A.E., Glazkov A.T., Vorotyntsev M.A. (2019). Electrochemically Driven Evolution of Br-Containing Aqueous Solution Composition. J. Electroanal. Chem..

[53] Yao Y., Lei J., Shi Y., Ai F., Lu Y.C. (2021). Assessment Methods and Performance Metrics for Redox Flow Batteries. Nat. Energy.

[54] Goulet M.-A., Aziz M. (2018). J Flow Battery Molecular Reactant Stability Determined by Symmetric Cell Cycling Methods. J. Electrochem. Soc..

[55] Bien H.-S., Stawitz J., Wunderlich K. (2000). Anthraquinone Dyes and Intermediates. Ullmann’s Encycl. Ind. Chem..

[56] Wiberg C., Carney T.J., Brushett F., Ahlberg E., Wang E. (2019). Dimerization of 9,10-anthraquinone-2,7-Disulfonic acid (AQDS). Electrochim. Acta.

[57] Carney T.J., Collins S.J., Moore J.S., Brushett F.R. (2017). Concentration-Dependent Dimerization of Anthraquinone Disulfonic Acid and Its Impact on Charge Storage. Chem. Mater..

[58] Yang Z., Tong L., Tabor D.P., Beh E.S., Goulet M.A., De Porcellinis D., Aspuru-Guzik A., Gordon R.G., Aziz M.J. (2018). Alkaline Benzoquinone Aqueous Flow Battery for Large-Scale Storage of Electrical Energy. Adv. Energy Mater..

[59] Mao J., Ruan W., Chen Q. (2020). Understanding the Aqueous Solubility of Anthraquinone Sulfonate Salts: The Quest for High Capacity Electrolytes of Redox Flow Batteries. J. Electrochem. Soc..

[60] Tong L., Chen Q., Wong A.A., Gómez-Bombarelli R., Aspuru-Guzik A., Gordon R.G., Aziz M.J. (2017). UV-Vis Spectrophotometry of Quinone Flow Battery Electrolyte for: In Situ Monitoring and Improved Electrochemical Modeling of Potential and Quinhydrone Formation. Phys. Chem. Chem. Phys..

[61] Arenas L.F., Ponce de León C., Walsh F.C. (2017). Engineering Aspects of the Design, Construction and Performance of Modular Redox Flow Batteries for Energy Storage. J. Energy Storage.

[62] Sun C.-Y., Znahg H. (2019). Investigation of Nafion series membranes on the performance of iron-chromium redox flow battery. Int. J. Energy Res..

[63] Loktionov P., Kartashova N., Konev D., Abunaeva L., Antipov A., Ruban E., Terent’ev A., Gvozdik N., Lyange M., Usenko A. (2021). Fluoropolymer impregnated graphite foil as a bipolar plates of vanadium flow battery. Int. J. Energy Res..

[64] Modestov A., Kartashova N., Pichugov R., Petrov M., Antipov A., Abunaeva L. (2022). Bromine Crossover in Operando Analysis of Proton Exchange Membranes in Hydrogen−Bromate Flow Batteries. Membranes.

[65] Gandomi Y.A., Aaron D.S., Houser J.R., Daugherty M.C., Clement J.T., Pezeshki A.M., Ertugrul T.Y., Moseley D.P., Mench M.M. (2018). Critical Review—Experimental Diagnostics and Material Characterization Techniques Used on Redox Flow Batteries. J. Electrochem. Soc..

[66] Lu M.Y., Deng Y.M., Yang W.W., Ye M., Jiao Y.H., Xu Q. (2020). A Novel Rotary Serpentine Flow Field with Improved Electrolyte Penetration and Species Distribution for Vanadium Redox Flow Battery. Electrochim. Acta.

[67] Houser J., Pezeshki A., Clement J.T., Aaron D., Mench M.M. (2017). Architecture for Improved Mass Transport and System Performance in Redox Flow Batteries. J. Power Sources.

[68] Dennison C.R., Agar E., Akuzum B., Kumbur E.C. (2016). Enhancing Mass Transport in Redox Flow Batteries by Tailoring Flow Field and Electrode Design. J. Electrochem. Soc..

[69] Huang Z., Mu A., Wu L., Wang H. (2021). Vanadium Redox Flow Batteries: Flow Field Design and Flow Rate Optimization. J. Energy Storage.

[70] Batchelor-McAuley C., Li Q., Dapin S.M., Compton R.G. (2010). Voltammetric Characterization of DNA Intercalators across the Full PH Range: Anthraquinone-2,6-Disulfonate and Anthraquinone-2-Sulfonate. J. Phys. Chem. B.

[71] Gamboa-Valero N., Astudillo P.D., González-Fuentes M.A., Leyva M.A., Rosales-Hoz M.D.J., González F.J. (2016). Hydrogen Bonding Complexes in the Quinone-Hydroquinone System and the Transition to a Reversible Two-Electron Transfer Mechanism. Electrochim. Acta.

[72] Huskinson B., Marshak M.P., Gerhardt M.R., Aziz M.J. (2014). Cycling of a Quinone-Bromide Flow Battery for Large-Scale Electrochemical Energy Storage. ECS Trans..

[73] Li G., Jia Y., Zhang S., Li X., Li J., Li L. (2017). The Crossover Behavior of Bromine Species in the Metal-Free Flow Battery. J. Appl. Electrochem..

