# Supplementary material for: Mixture of Anthraquinone Sulfo-Derivatives as an Inexpensive Organic Flow Battery Negolyte: Optimization of Battery Cell
Source: Membranes (Basel). 2022 Sep 21;12(10):912. doi: 10.3390/membranes12100912 (PMC9607404; doi:10.3390/membranes12100912)
Supplement: Supplementary file 1 [file membranes-12-00912-s001.zip › membranes-1909063-supplementary.pdf]

Supplementary Information

Table S1. Main characteristics of redox behavior obtained by CV.

Substance	Scan rate (mV s^{-1})	Peak-to-peak separation[a], dE (mV)	Half-wave potential[b], $E_{1/2}$ (V)	Peak current ratio[c], I_c/I_a
ASM	50	54	0.018	0.85
	100	59	0.017	0.83
	250	73[d]	0.011	0.84
	500	98	0.005	0.86
	1000	103	0.005	0.90
2,7-AQDS	50	32	0.019	0.95
	100	36	0.019	0.94
	250	36	0.017	0.97
	500	29	0.013	1.01
	1000	27	0.012	1.03
2,6-AQDS	50	26	0.022	0.95
	100	27	0.022	0.94
	250	21	0.021	0.97
	500	20	0.019	1.01
	1000	23	0.012	1.03
2-AQS	50	24	-0.027	1.06
	100	28	-0.027	1.02
	250	23	-0.030	0.99
	500	23	-0.032	1.00
	1000	27	-0.031	0.94

[a] $dE = E_a - E_c$, where E_a/E_c – anodic/cathodic peak potentials

[b] $E_{1/2} = \frac{E_a + E_c}{2}$

[c] where I_a/I_c – anodic/cathodic peak current

[d] ASM anodic wave for scan rates over 50 mV s^{-1} was treated as a single peak

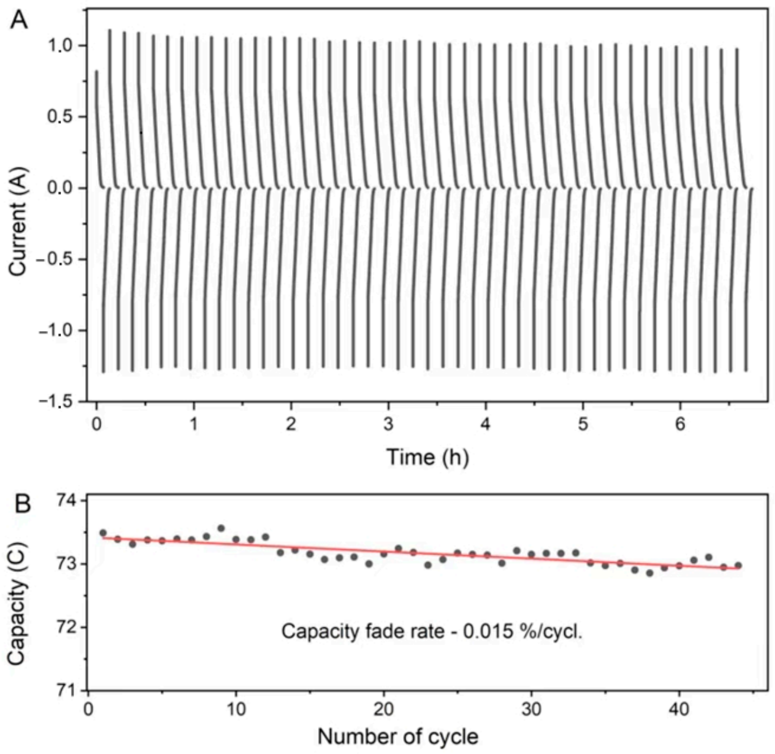

Figure S1. (A) Charge-discharge curves for constant current symmetrical cycling of ASM. Applied voltage – 0.2. (B) Dependence of the discharge capacity on the cycle number
